# Imputation Strategies Under Clinical Presence: Impact on Algorithmic Fairness

**Published:** 2022

**Authors:** Vincent Jeanselme, Maria De-Arteaga, Zhe Zhang, Jessica Barrett, Brian Tom

**Affiliations:** MRC Biostatistics Unit, University of Cambridge, Cambridge, UK; McCombs School of Business, University of Texas at Austin, Austin, USA; Rady School of Management, University of California, San Diego, USA; MRC Biostatistics Unit, University of Cambridge, Cambridge, UK; MRC Biostatistics Unit, University of Cambridge, Cambridge, UK

**Keywords:** Clinical Presence, Fairness, Imputation

## Abstract

Biases have marked medical history, leading to unequal care affecting marginalised groups. The patterns of missingness in observational data often reflect these group discrepancies, but the algorithmic fairness implications of group-specific missingness are not well understood. Despite its potential impact, imputation is too often an overlooked preprocessing step. When explicitly considered, attention is placed on overall performance, ignoring how this preprocessing can reinforce groupspecific inequities. Our work questions this choice by studying how imputation affects downstream algorithmic fairness. First, we provide a structured view of the relationship between clinical presence mechanisms and groupspecific missingness patterns. Then, through simulations and real-world experiments, we demonstrate that the imputation choice influences marginalised group performance and that no imputation strategy consistently reduces disparities. Importantly, our results show that current practices may endanger health equity as similarly performing imputation strategies at the population level can affect marginalised groups differently. Finally, we propose recommendations for mitigating inequities that may stem from a neglected step of the machine learning pipeline.

## 1 Introduction

Machine learning models for healthcare often rely on observational data. At the core of observational data generation is a complex interaction between patients and the healthcare system, which we refer to as *clinical presence* (Jeanselme et al., 2022). Each observation, from orders of laboratory tests to treatment decisions, reflects access to medical care, patients’ medical states, and also practitioners’ expertise and potential biases. Historically, healthcare access, treatment and outcomes have been marked by inequalities (Chen et al., 2021; Freeman and Payne, 2000; Jeanselme et al., 2021; Kim et al., 2016; Norris and Nissenson, 2008). For instance, Price-Haywood et al. (2020) hypothesised that the disproportionate mortality rate from Covid-19 among Black patients can, in part, be explained by longer waiting times before accessing care.

Clinical presence patterns can, therefore, reflect disparities. Specifically, observation and missingness can vary across groups. Developing machine learning models on these data raises ethical concerns about automating and reinforcing injustices.

Current practices for handling missing data often rely on imputing data with overall performance in mind (Emmanuel et al., 2021), without consideration of the algorithmic fairness consequences associated with this choice. Despite the risk of aggravating inequities reflected in group-specific missingness patterns, the effect of this imputation step remains understudied. In this work, we explore the impact of imputation on data imprinted by group-specific missingness patterns emerging from medical practice and historical biases. First, we identify scenarios of clinical presence that could result in group-specific missingness patterns, grounded on historical evidence of these phenomena in medicine. Then, we explore the downstream impact on group performance of standard imputation strategies on simulated data affected by this *clinical missingness*. Finally, we study group performances of different imputation strategies in real-world data.

This work provides empirical evidence that machine learning pipelines differing solely in their handling of missingness may result in distinct performance gaps between groups, even when population performances present no difference. The choice of imputation strategy may therefore impact performance in a way that reinforces inequities against historically marginalised groups. Moreover, our experiments show that no imputation strategy consistently outperforms the others and current recommendations may harm marginalised groups. Finally, we emphasise the relevance of this analysis by providing real-world evidence of clinical missingness patterns and echo the previous results in the MIMIC III dataset.

## 2 Related work

This work explores the link between missingness and algorithmic fairness in machine learning for healthcare. In this section, we review related literature across domains.

### 2.1 Clinical missingness

Clinical missingness is a medical expression of the well-studied missingness patterns (Little and Rubin, 2019): *Missing Completely At Random* (MCAR) — random subsets of patients and/or covariates are missing, *Missing At Random* (MAR) — missing data patterns are a function of observed variables, and *Missing Not At Random* (MNAR) — missing patterns depend on unobserved variables or the missing values themselves.

Traditional statistical models are not adapted to handle missing covariates. Consequently, practitioners may rely on single imputation strategies such as mean, median, nearest neighbours (Batista et al., 2002; Bertsimas et al., 2021) or the preferred multiple imputation methods (Newgard and Lewis, 2015; Rubin, 2004; White et al., 2011). Typically, these imputation approaches assume MCAR and/or MAR patterns. They may be ill-adapted to handle informative missingness, particularly as MNAR and MAR are non-identifiable from observational data alone and require domain expertise for adequate modelling. The recommended strategy to tackle this non-identifiability issue is to control the imputation model on additional covariates to render the MAR assumption more plausible (Haukoos and Newgard, 2007). Our work shows the potential shortcomings of this covariate-adjusted imputation strategy under group-specific missingness patterns.

### 2.2 Algorithmic fairness in medicine

The risk of reinforcing historical biases is of critical concern in medicine, where inequalities can have life-threatening implications. Measuring and mitigating this risk is the aim of algorithmic fairness (Chouldechova and Roth, 2020). In this paper, we follow the ‘*equal performance*’ group definition of algorithmic fairness (Rajkomar et al., 2018), which evaluates if the model performs comparably across groups (Chouldechova et al., 2018; Flores et al., 2016; Noriega-Campero et al., 2019).

#### Definition 1 (Equal Performance)

*A pipeline p is fairer than another q with regard to group g if its performance gap is the smallest, i.e*. |Δ*_g_*(*p*)| < |Δ_*g*_(*q*)| *with* Δ_*g*_(*p*) ≔ *d*(*p*({*X_i_*}*G_i_*=*g*)) – *d*(*p*({*X_i_*}*G_i_*≠*g*)) *for some performance metric d, a pipeline p and (*X_i_, G_i_*), the covariates and associated group for patient i*.

This metric has been leveraged to quantify models’ impact on algorithmic fairness in medicine (Chen et al., 2018, 2019; Pfohl et al., 2019; Seyyed-Kalantari et al., 2020; Zhang et al., 2020). For instance, Seyyed-Kalantari et al. (2020) demonstrates X-ray classifiers’ performance gap between marginalised groups. However, the link between imputation and algorithmic fairness has received limited attention despite the risk of clinical missingness disparities. Our work aims to fill this gap.

### 2.3 Algorithmic fairness and missingness

As a community, we need to understand how to best handle clinical missingness when imprinted by biases. Martínez-Plumed et al. (2019); Fricke et al. (2020) show that mean imputation presents better fairness properties compared to complete case analysis. These works focus on one imputation strategy and ignore the potential variability of the impact of different strategies. Closer to our work, Zhang and Long (2021) show that the choice of imputation may lead to different fairness gaps when enforcing synthetic missingness patterns. However, these works do not discuss how the different missingness patterns may arise in medicine, and how a specific group may be impacted differently by different imputation strategies. In our work, we study different missingness patterns that may arise as a result of the data-generating process in healthcare. Finally, Ahmad et al. (2019); Ghassemi et al. (2020); Rajkomar et al. (2018) describe multiple challenges linked to medical data, among which they state that historical biases may lead to missingness patterns that could impact fairness, but they do not empirically study this. While informative missingness has recently received revived attention (Jeanselme et al., 2022; Getzen et al., 2022), no work has studied its potential association with fairness. Our work aims to address these gaps in the literature by demonstrating the existence of this problem, characterising different types of group-specific missingness patterns in medicine, and exploring the impact of different imputation strategies under different clinical presence scenarios. In addition to showing the impact of imputation choice on fairness gaps, we highlight that the *same imputation strategy* may benefit a group under one missingness pattern but hurt this same group in another. Importantly, we also show that a given group may benefit under one imputation and suffer under another imputation in the same setting, even if the two strategies perform identically at the population level. These are novel findings that invite practitioners to perform careful sensitivity analysis of imputation choice on fairness gaps.

## 3 Clinical missingness scenarios

**Figure 1:**
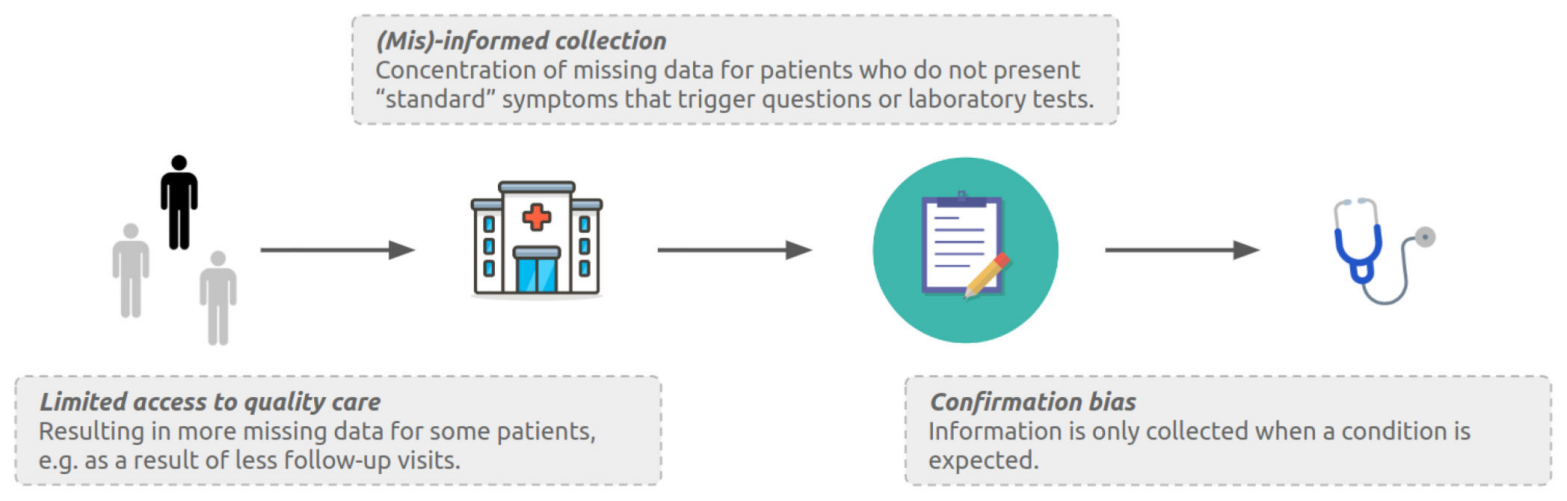
Examples of group-specific clinical presence mechanisms.

This section shows how group-specific missingness can result from clinical presence. Figure 1 introduces the following scenarios:

### Limited access to quality care (S1)

When certain groups do not have access to the same health services, this results in more missing covariates for these groups.

Socioeconomic factors resulting from structural injustices (Barik and Thorat, 2015; Nelson, 2002; Szczepura, 2005; Yearby, 2018) such as insurance, work schedule flexibility, distance to hospitals (Barik and Thorat, 2015) or mobility, result in inconsistent medical history (Gianfrancesco et al., 2018), additional waiting time before looking for care (Weissman et al., 1991), avoidance of preventing care (Smith et al., 2018), and limited access to advanced diagnostic tools (Lin et al., 2019). This diminished access to care is potentially reflected as missing data. For instance, patients may have no annual checkup data if their insurance does not cover or encourage this service.

### (Mis)-informed collection (S2)

Often, medical research has focused on a subset of the population. The resulting guidelines may be ill-adapted to other groups and relevant covariates may be missing due to standard recommendations.

Historically researchers focused on (perceived) highest-risk groups: breast cancer predominantly studied in women (Arnould et al., 2006; Giordano, 2018), cardiovascular disease in men (Vogel et al., 2021), skin cancers in whiter skins (Gloster Jr and Neal, 2006), and autism in men (Gould and Ashton-Smith, 2011). Resultant medical practices and guidelines target these groups. However, substantial evidence shows the prevalence of these diseases among other groups. Stemming from biological differences, different groups may present different symptoms and expressions for the same condition. The difference in disease expression and the absence of adapted tests result in missing covariates necessary to identify the disease. For instance, screening recommendations may only be prescribed conditioned on observation of “standard” symptoms. If the symptoms considered are not the expected disease expression for a marginalised subgroup, this will result in more missing screening procedures for this group.

### Confirmation bias (S3)

Practitioners collect data based on expertise and informative proxies that are not recorded, e.g. patient feeling unwell.

For instance, practitioners may record the value of a test only if they suspect it will be abnormal. The literature presents evidence of this phenomenon where the presence of a specific medical test is more informative of the outcome than the test result itself (Agniel et al., 2018; Sisk et al., 2020). Wells et al. (2013) also suggest that missing laboratory tests correspond to healthy results, e.g. doctors do not collect or record data if they are irrelevant. Similarly, sicker patients present more complete data (Rusanov et al., 2014; Sharafoddini et al., 2019; Weiskopf et al., 2013).

### Formalisation

Consider two covariates (*X*_1_, *X*_2_) influenced by the underlying condition *Y* and the group membership *G*. Note that the disease prevalence may also depend on G. One covariate X_1_ is observed for all patients, while X_2_ is potentially missing. Following the notations from Mohan and Pearl (2021), let *O*_2_ be the indicator of observation of *X*_2_ such that the observed value is defined as:

$$X_{2}^{*}=\{\begin{array}{ll} \emptyset & \text{if}\;O_{2}=0\\ X_{2} & \text{otherwise} \end{array}$$

In (S1), G informs *O*_2_ because of group socioeconomic differences. In (S2) and (S3), G impacts the observation process through group-specific disease expression. While the influence of medical covariates on the missingness patterns characterises both (S2) and (S3), (S2) describes how guidelines may depend on observed covariates, whereas (S3) reflects how the observation process may depend on X_2_ itself or unobservable covariates correlated with *X*_2_. For instance, (S2) may consist of a guideline recommending to measure *X*_2_ if *X*_1_ is within a given range. However, if a patient is a member of a group for which X_1_ is not informative—or for which the informative range is different—X_2_ might not be observed as *X*_1_ is not in the guideline test-triggering range. This may lead to more missing data for *X*_2_ in the group with different characteristics for *X*_1_. (S3) differs as practitioners would *record* the value of *X*_2_ only if this one is abnormal.

**Figure 2:**
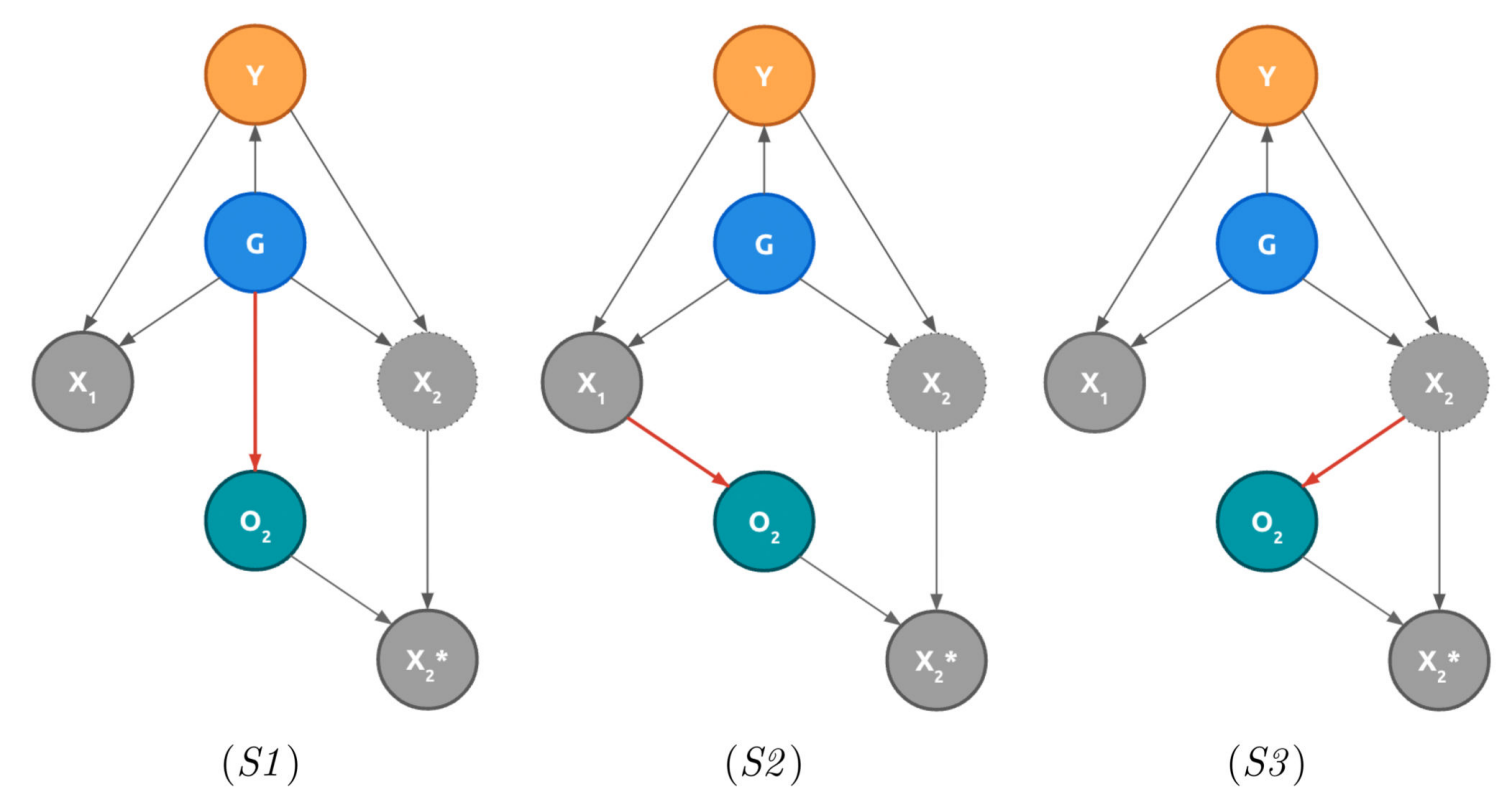
Directed Acyclic Graphs (DAGs) associated with the identified clinical missingness scenarios. Full circled covariates are observed, dotted ones unobserved. *Y* is the condition, *G*, the group membership, *X*_1_ and *X*_2_ the two covariates. *O*_2_ is the observation process associated to *X*_2_. Red dependencies underline the differences between scenarios.

These dependencies result in three distinct patterns between missingness, group and covariates, summarised with directed acyclic graphs (DAGs) in Figure 2.

## 4 Experiments

In this section, we explore how the choice of imputation affects group-specific performance, and potentially reinforces disparities in data marked by clinical missingness. We first present simulation studies in which we enforce specific missingness patterns. This analysis allows us to control clinical missingness patterns and measure the potential impact of imputation on algorithmic fairness. We accompany these results with real-world evidence of group-specific missingness patterns and show the impact of different imputation strategies on marginalised group performance. For reproducibility, all experiments’ code is available on Github^1^.

### 4.1 Datasets

Assume a population of *N* patients with associated covariates *X*, marginalised group membership *G*, and outcome of interest *Y*.

#### Simulation

**Figure 3:**
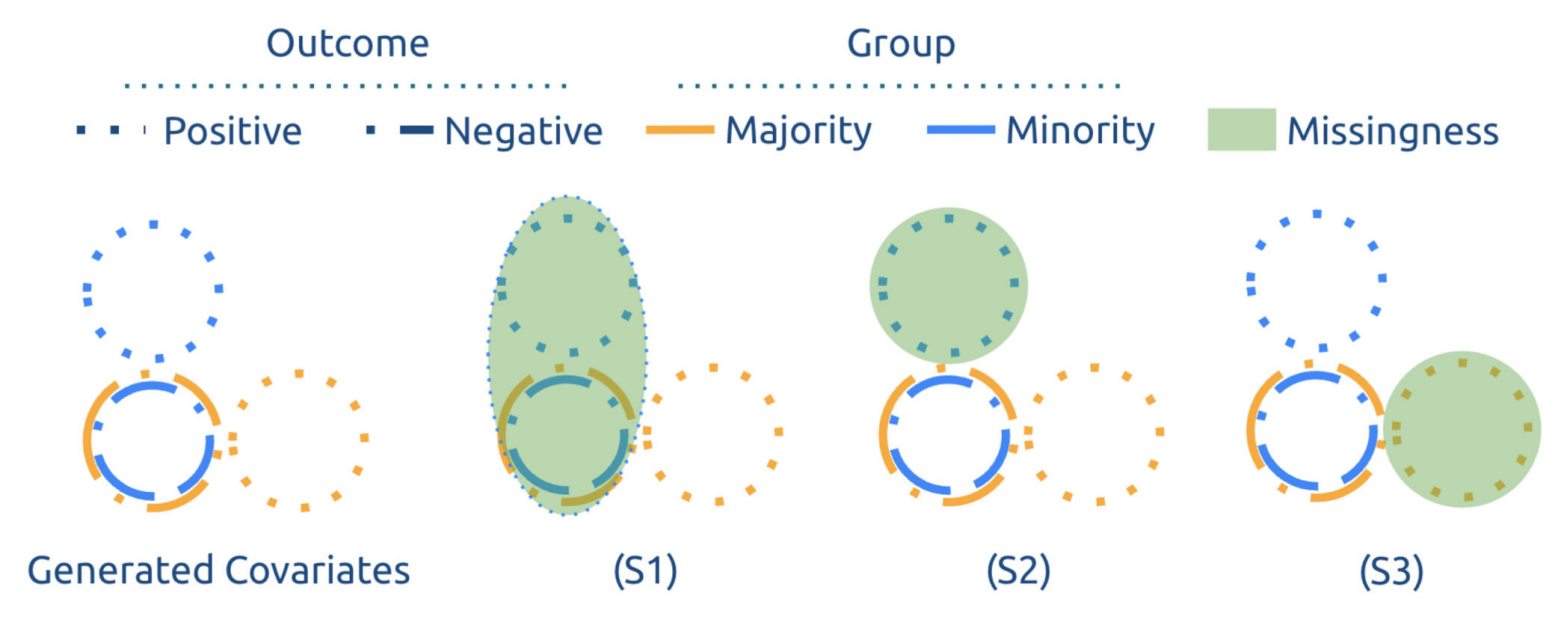
Graphical summary of clinical missingness enforcement in the simulation experiments. Note that our simulations’ choices result in missingness in the marginalised group only in (S1) and (S2), but in the majority only in (S3).

We introduce a bidimen-sional (*X* ∈ ℝ^2^) synthetic population (*N* = 10,100) divided into two groups (*G* ∈ {0,1}), and assume the marginalised group is a minority in the population with ratio 1:100. These groups differ in disease expression, i.e. positive cases across groups differ in how they express the disease. Then clinical missingness patterns are enforced on the second dimension *X*_2_ following the scenarios introduced in Section 3. Figure 3 provides a graphical summary of how clinical missingness is enforced on the synthetic data. The associated predictive task is to classify between positives and negatives. (See Appendix A.1 for full data generation protocol reflecting the enforcement of the previously-introduced scenarios).

#### MIMIC III

The real-world analysis relies on the laboratory tests from Medical Information Mart for Intensive Care (MIMIC III) dataset (Johnson et al., 2016). Following data harmonisation (Wang et al., 2020), we select adults who survived 24 hours or more after admission to the intensive care unit, resulting in a set of 36,296 patients sharing 67 laboratory tests. The goal is to predict short-term survival (7 days after the observation period — Y) using the most recent value of each laboratory test observed in the first 24 hours of observation (*X*). We select short-term survival as it is a standard task in the machine learning literature (Jeanselme et al., 2022; Nagpal et al., 2021; Tsiklidis et al., 2022; Xu et al., 2019) and the associated labels are less likely to suffer from group-specific misdiagnosis, and, therefore, disentangles our analysis from potential biases in labelling (Chen et al., 2020). In practice, deploying this model could be used for care prioritisation of patients with predicted elevated risk.

### 4.2 Handling missing data

The simulation and MIMIC III datasets present missing data that are traditionally imputed for analysis. We consider the following common imputation strategies:

#### Single median imputation (Median)

Missing data are replaced by the population median of each covariate. Due to its straightforward implementation, this methodology remains predominant in the literature despite known shortcomings (Rubin, 1976; Sinharay et al., 2001; Crawford et al., 1995).

#### Multiple Imputation using Chained Equation (MICE)

Missing data are iteratively drawn from a regression model built over all other available covariates after median initialisation. This approach is repeated *I* times with an associated predictive model for each imputed draw. At test time, the same imputation models generate I imputed points for which models’ predictions are averaged. MICE is recommended in the literature (Janssen et al., 2010; Newgard and Haukoos, 2007; Wood et al., 2004; Zhou et al., 2001; White et al., 2011) as it quantifies the uncertainty associated with missingness. In the experiments, we used 10 iterations repeated 10 times resulting in *I* =10 datasets with associated predictive models.

#### Group MICE

The previous MICE methodology assumes a MAR mechanism. To make this assumption more plausible, Haukoos and Newgard (2007) recommend the addition of potentially informative covariates. In our experiment, we, therefore, rely on both group membership and covariates for imputing the missing data ($\tilde{X}∼X,G$ with $\tilde{X}$ representing the imputed covariates).

#### Group MICE Missing

Encoding missingness has been shown to improve performance when the patterns of missingness are informative (Groenwold, 2020; Lipton et al., 2016; Saar-Tsechansky and Provost, 2007; Sperrin et al., 2020). As clinical missingness can contain informative patterns (Jeanselme et al., 2022; Lipton et al., 2016), we concatenate missingness indicators to the imputed data from Group MICE (Appendix A explores the concatenation of missing indicators with the other strategies).

### 4.3 Experimental setting

After imputation, each pipeline relies on a logistic regression model — a pillar in medicine (Nick and Campbell, 2007; Goldstein et al., 2017) — to discriminate between positive and negative cases $\left(Y∼\tilde{X}\right)$.

Adopting the *equal performance across groups* definition (Rajkomar et al., 2018) of algorithmic fairness, we measure each pipeline’s discriminative performances for the different groups. We use the Area Under the Curve for the Receiver Operating Characteristic curve (AUC - ROC, i.e. *d* in Section 2.2) as proposed in Röösli et al. (2022); Larrazabal et al. (2020); Zhang et al. (2022).

This metric quantifies algorithmic fairness but does not quantify how deployment can hurt subgroups at a fixed threshold on the predicted risk. In the MIMIC III study, we measure the False Negative Rate (FNR) assuming the availability of priority care for 30% of the population (sensitivity to this threshold is presented in Appendix A.2). In the 30% highest-risk population, we measure the prioritisation — the group-specific proportion of patients who would receive care under this policy — and misclassification rates in the groups of interest. In this setting, FNR corresponds to the non-prioritisation of high-risk patients. The gap in FNR between groups answers the question: how marginalised groups would be incorrectly deprioritized? Additional experimental design descriptions and results are provided in Appendix A.

## 5 Results

This section presents the insights obtained through both simulations and real-world experiments.

### 5.1 Simulations

**Figure 4:**
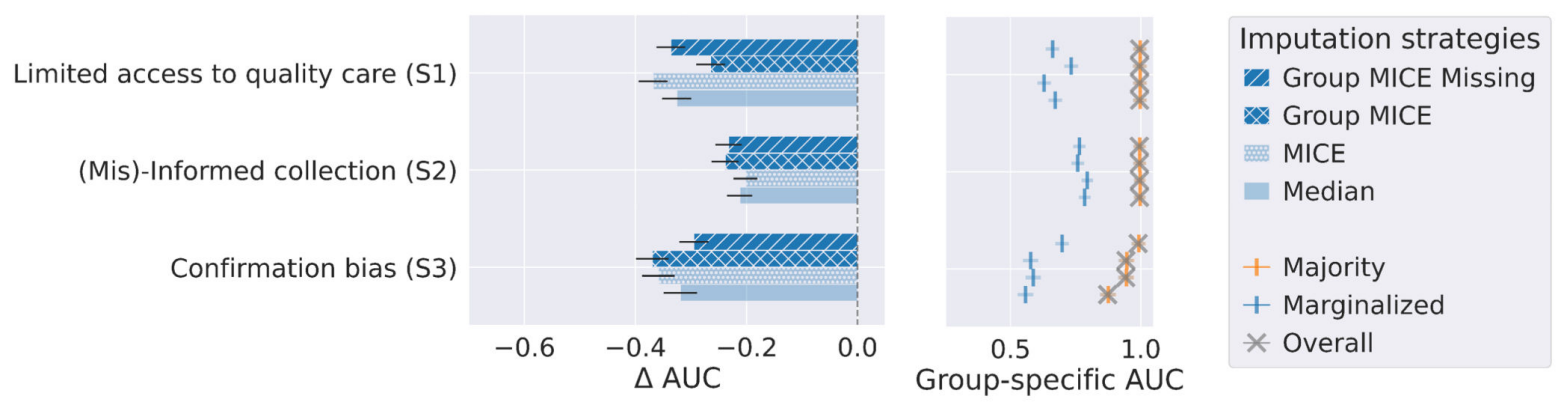
AUC performance gaps Δ and group-specfic AUCs across scenarios on 100 synthetic experiments. If Δ < 0, the marginalised group has worse AUC than the majority.

We conduct 100 simulations in which the three clinical presence scenarios are independently enforced. We apply the imputation strategies described in Section 4.2 and train a logistic regression with l2 penalty (λ = 1). Results are computed on a 20% test set and averaged over the 100 simulations. Figure 4 presents the AUC gap (Δ defined in Section 2.2) between the majority and the minority, and group-specific AUCs.

#### Insight 1: Equally-performing imputation strategies at the population level can result in different marginalised group performances

Consider (S1), all imputation methodologies result in similar population AUCs, as shown by the grey dots. However, note how the AUC evaluated on the marginalised group presents a gap of 0.1 between MICE and Group MICE. This phenomenon is explained by how imputation strategies result in different imputed covariate distributions. The logistic regressions built on these imputed data would weigh covariates differently and then have different predicted values.

#### Insight 2: No strategy consistently outperforms the others across clinical presence scenarios

Population-level performances remain stable between Group MICE and MICE over all scenarios, but these strategies have contrasting marginalised group AUCs. Importantly, Group MICE should be preferred in (S1) as it minimises the performance gap. For the same reason, MICE should be used in (S2), whereas both methodologies present inconclusive fairness differences in (S3). While this result is specific to this simulation, this exemplifies how no methodology consistently reduces the performance gap across groups.

#### Insight 3: Current recommendation of leveraging additional covariates to satisfy MAR assumption, or using missingness indicators can harm marginalised group’s performance

Note how Group MICE presents *worse* performance than MICE in (S2). The recommendation of including additional covariates to make the MAR assumption more plausible is not always suitable as it may add *noise* and lead to poorer performance. In another example, see how the model considering missingness provides an edge in (S3) compared to Group MICE but hurts performance in (S1). This observation reinforces the necessity of measuring the performance sensitivity to imputation. Additionally, it underlines how understanding the missingness process is essential to control for relevant covariates.

### 5.2 MIMIC III

In this real-world experiment, we consider groups defined by the following attributes: ethnicity (Black vs non-Black), sex (female vs male), and insurance (publicly vs privately insured). Table 1 shows the number of orders and the number of distinct laboratory tests (out of the 67 possible tests) performed during the first-day post-admission for each subgroup. This last number reflects the missingness of the vector used for prediction.

**Figure 5:**
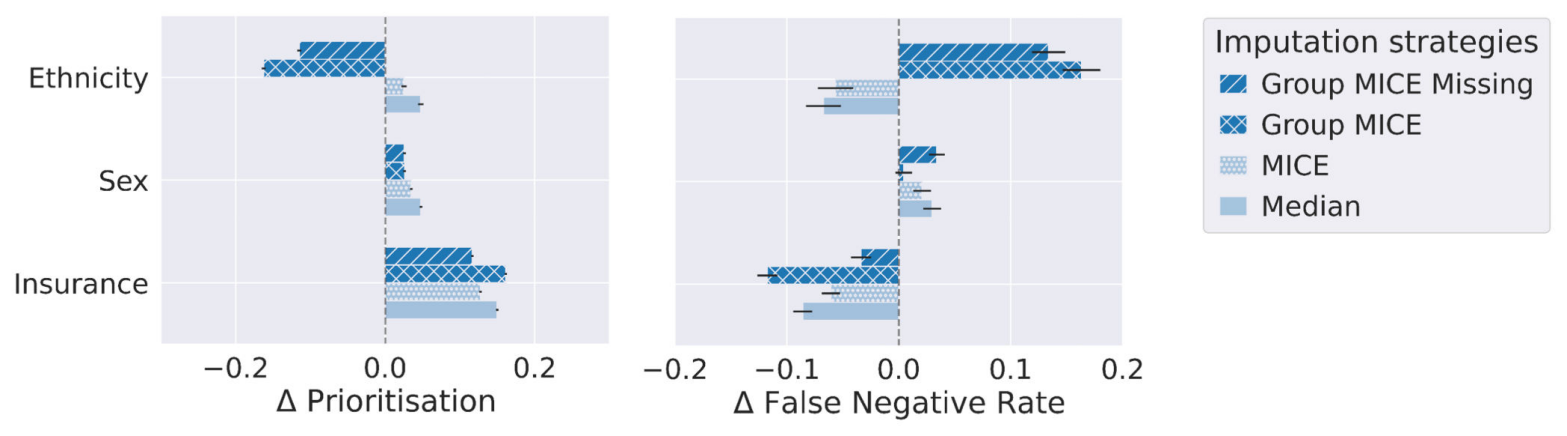
Prioritisation performance gaps Δ across marginalised groups in MIMIC III experiment. If Δ > 0, the marginalised group has a larger value of the given metric than the rest of the population.

For this experiment, patients are split into three sets: 80% for training, 10% for hyperparameter tuning and 10% for testing. We perform a l2 penalty search for the logistic regression among λ ∈ [0.1,1,10,100]. Table 2 presents predictive performances at the population level averaged on the bootstrapped test set over 100 iterations. Assuming capacity for additional care for the 30% highest risk, we explore care prioritisation. Figure 5 displays our main results: the gaps in prioritisation and the false negative rates stratified by groups of interest under the different imputation strategies.

#### Insight 4: Real-world data presents group-specific clinical presence patterns

While the causes of clinical missingness *cannot* be distinguished from observational data alone, one can observe evidence of non-random missingness patterns in the MIMIC III dataset, as shown in Table 1. Specifically, note the larger number of orders for patients who die during their stay compared with the ones who survive. This pattern is consistent with a possible *confirmation bias* scenario (S3), if doctors are monitoring sicker patients more closely. Another example of non-random missingness is that there are fewer test orders for female, Black, and publicly insured patients, but little difference in the diversity of tests prescribed. While this may be explained by the underlying conditions or other medically relevant factors, the combination of similar diversity of tests but less frequent observations results in a less up-to-date patient’s health status for modelling. Thus, even though the cause of testing differences is unclear, these observations show the connection between testing patterns, group membership, and outcomes. This real-world evidence of non-random missingness patterns among subgroups of patients raises concerns about increasing inequities if the fairness implications of imputation methods are not considered.

#### Insight 5: Marginalised groups can benefit or be harmed by equally performing imputation strategies at the population level

Note how MICE and Group MICE perform similarly at the population level in Table 2, but present different performances for marginalised groups (see Figure 5). Consider the ethnicity split: these methodologies have opposite consequences on Black patients. MICE would result in more care for Black patients and a smaller gap in FNR. By contrast, Group MICE would halve prioritisation and double the FNR gap in favour of non-Black patients. Crucially, this difference solely results from the imputation strategy adopted in these two pipelines.

#### Insight 6: Different marginalised groups may be impacted oppositely by the same imputation strategy

Female and publicly insured patients have higher prioritisation rates under all imputation methods. However, these groups show opposite gaps in their FNR compared to their counterparts (men and privately insured patients): women have more false negative cases missed while those publicly insured have fewer false negatives.

In another case of opposite impacts of imputation, Group MICE presents the smallest FNR performance gap for sex, but the largest gaps for both ethnicity and insurance. Group MICE also results in better FNR performance for publicly insured but worse for Black patients. This observation underlines the importance of identifying marginalised groups in development and deployment populations. The optimal trade-off between group and population performances, and between marginalised groups, needs to be considered as different pipelines could have opposite impacts.

## 6 Discussion

This paper is motivated by how interactions between patients and the healthcare system can result in group-specific missingness patterns. We show that resultant inequities in clinical missingness can impact downstream algorithmic fairness under different imputation strategies. This analysis demonstrates that no imputation strategy consistently provides better performances for marginalised groups. In particular, a model providing an edge in one setting can underperform in another, or even harm a different group. Moreover, the experiments conducted using the MIMIC-III dataset demonstrate the relevance of the identified problem as more than a merely theoretical concern, showing that it is present in a widely used electronic health record dataset.

Note that our work does not claim that the specific patterns we observe will necessarily be present in other datasets. As we have emphasised, different combinations of missingness processes may lead to different fairness gaps and interactions between imputation and group performance. It may even lead to equal fairness performance of all imputation strategies, but one cannot know this a priori.

Learning from medical data without sufficient attention to the potential entanglement of clinical missingness and historical biases could reinforce and automatise inequities, and further harm historically marginalised groups. This work calls for caution in the use of imputation to reach health equity. We invite practitioners to:

- Record protected attributes and identify marginalised groups.
- Explore the practitioner-patient interaction process to identify clinical missingness disparities.
- Report the assumptions made at each stage of the pipeline.
- Perform sensitivity analysis on imputation to understand its impact on algorithmic fairness.

Future work will theoretically define in which settings the presented results stand and how model choice could mitigate discrepancies in the missingness patterns. Moreover, clinical missingness is only one dimension of how clinical presence shapes the data-generating process. The temporality and irregularity of medical time series may convey group-specific disparities that machine learning methods may amplify.

## Supplementary Material

Appendix A

## Figures and Tables

**Table 1:** Mean (std) number of orders and observed tests performed during the first post-admission stratified by marginalised group and outcomes.

	Orders		Distinct tests	
Alive^+^	5.68 (4.64)	*	40.80 (6.73)	*
Dead^+^	7.57 (5.44)		37.22 (7.50)	
Black	5.24 (4.08)	*	40.94 (6.94)	*
Other	5.86 (4.77)		40.52 (6.84)	
Female	5.54 (4.45)	*	40.75 (6.89)	*
Male	6.03 (4.91)		40.41 (6.80)	
Public	5.67 (4.57)	*	40.46 (6.76)	*
Private	6.11 (5.01)		40.75 (7.01)	

+ By the 8*^th^* day after admission.

* Significant t-test p-value (< 0.001).

**Table 2:** Predictive performance under different imputation strategies. Mean (std) computed on the test set bootstrapped 100 times.

	AUC ROC
Group MICE Missing	**0.786** (0.009)
Group MICE	0.738 (0.012)
MICE	0.742 (0.012)
Median	0.748 (0.011)

## Footnotes

1
https://github.com/Jeanselme/ClinicalPresenceFairness
