# Supplementary material for: Imputation Strategies Under Clinical Presence: Impact on Algorithmic Fairness
Source: Proc Mach Learn Res. Author manuscript; Available in PMC 2023 Jan 3. (PMC7614014)
Supplement: Appendix A [file EMS158739-supplement-Appendix_A.pdf]

Appendix A. Experiments

This section provides additional details on the experimental design.

A.1. Simulation study

Data Generation. The proposed synthetic population consists of 10,000 points for the majority group and 100 for the marginalised group resulting in a sample size of $N = 10,100$ with a ratio of 100:1. Each individual is represented in this dataset as a pair of covariates, i.e. $X \in \mathbb{R}^2$. For each group, 50% presents the condition, i.e. $\mathbb{P}(Y_i = 0) = 0.5$. Negatives are drawn from the normal distribution $\mathcal{N}((0,0), 0.25)$. The disease characterisation, i.e. the boundary between positive and negatives, differs between groups with positive from the majority (resp. the marginalised group) sampled from $\mathcal{N}((1,0), 0.25)$ (resp. $\mathcal{N}((0,1), 0.25)$). Figure 6 shows the density distribution of the generated population.

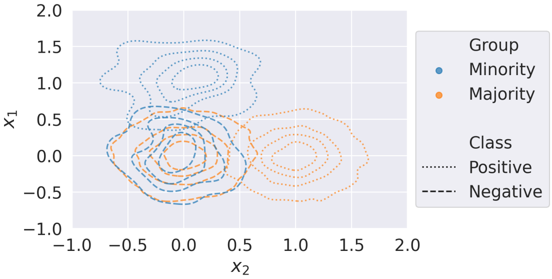

Figure 6: Density distributions of the generated population.

Missingness. In this synthetic population, 50% of the dimension X_2 is removed in a given subgroup to enforce the three clinical presence scenarios. We enforce the following clinical missingness:

- Limited access to quality care (S1):

$$O_2 \mid [G = 1] \sim \mathcal{B}ern(0.5)$$

- (Mis)-informed collection (S2):

$$O_2 \mid [X_1 > 0.5] \sim \mathcal{B}ern(0.5)$$

- Confirmation bias (S3):

$$O_2 \mid [X_2 > 0.5] \sim \mathcal{B}ern(0.5)$$

With O_2 the observation indicator associated with X_2 and G, the group membership ($G = 1$ indicates a member of the marginalised group).

Modelling. We generate 100 datasets and enforce the different missingness patterns before running a logistic regression with an l2 penalty ($\lambda = 1$). Results are computed on the 20% test set and averaged over the 100 iterations with 95% confidence bounds reported.

Tabular results. Table 3 presents the AUC-ROC for the minority and majority groups and the different imputation strategies.

Missing Indicators. Similarly, Table 4 presents the AUC-ROC when a missing indicator is added for modelling (as proposed in Section 4.2). These results echos similar points to the main paper:

- State-of-the-art methodologies can perform similarly at the population level but harm marginalised groups differently as shown in (S1).
- No methodology consistently outperforms the others.
- Recommendation of adding missing indicators can hurt performance as MICE and Group MICE show in (S2).
- All methodologies benefit in (S3) in which the missingness is informative of the missing value itself.

Table 3: AUC-ROC divided by scenarios, group and imputation strategy - Mean (std) over 100 simulations.

		Imputation strategy		
Scenario	Group	Median	MICE	Group MICE
	Majority	0.997 (0.000)	0.997 (0.000)	0.997 (0.000)
(S1)	Minority	0.672 (0.026)	0.629 (0.026)	**0.733** (0.026)
	Population	0.995 (0.000)	0.995 (0.000)	0.995 (0.000)
	Majority	0.997 (0.000)	0.997 (0.000)	0.997 (0.000)
(S2)	Minority	0.785 (0.023)	**0.795** (0.022)	0.758 (0.024)
	Population	0.995 (0.000)	0.995 (0.000)	0.994 (0.000)
	Majority	0.876 (0.002)	0.945 (0.001)	0.947 (0.001)
(S3)	Minority	0.557 (0.030)	**0.587** (0.029)	0.577 (0.029)
	Population	0.873 (0.002)	0.942 (0.001)	0.943 (0.001)

Table 4: AUC-ROC divided by scenarios, group and imputation strategy with missing indicators - Mean (std) over 100 simulations.

		Imputation strategy with **missing indicator**		
Scenario	Group	Median	MICE	Group MICE
	Majority	0.997 (0.000)	0.997 (0.000)	0.997 (0.000)
(S1)	Minority	**0.684** (0.026)	0.641 (0.026)	0.661 (0.026)
	Population	0.995 (0.000)	0.995 (0.000)	0.994 (0.000)
	Majority	0.997 (0.000)	0.997 (0.000)	0.997 (0.000)
(S2)	Minority	**0.798** (0.022)	0.797 (0.021)	0.764 (0.024)
	Population	0.995 (0.000)	0.995 (0.000)	0.994 (0.000)
	Majority	0.993 (0.000)	0.992 (0.000)	0.992 (0.000)
(S3)	Minority	0.694 (0.026)	**0.700** (0.026)	0.698 (0.026)
	Population	0.990 (0.000)	0.989 (0.001)	0.989 (0.000)

A.2. MIMIC III

Dataset. After preprocessing (Wang et al., 2020) and standardisation, the MIMIC III dataset consists of $36,296$ patients with 67 different laboratory tests. Focusing on the three marginalised groups of interest, the population can be further divided into marginalised subgroups as presented in Figure 7. First, this real-world distribution justifies our simulation choice as group intersection might be underrepresented in a dataset and present different characteristics than the majority. Second, this underlines the problem of identifying marginalised groups as they can be impacted differently by the same model.

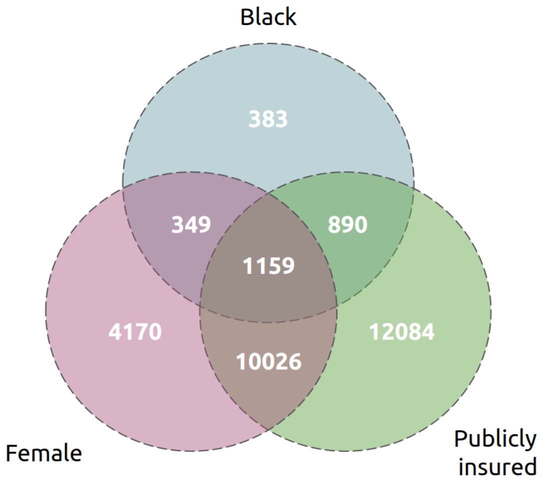

Figure 7: Venn diagram of the population distribution in the three marginalised groups.

Further clinical presence evidence. Table 1 shows the difference in testing at the end of the 24-hour observation period, one can study the temporal evolution of the testing procedure. Clinical presence is expressed not only in the missingness but in the temporality of the generative process (Jeanselme et al., 2022). Figure 8 displays the temporal evolution of the number of laboratory tests performed for both survivors and patients who die after the observation period. This motivates our future work on studying the impact of strategies handling irregular time series on algorithmic fairness.

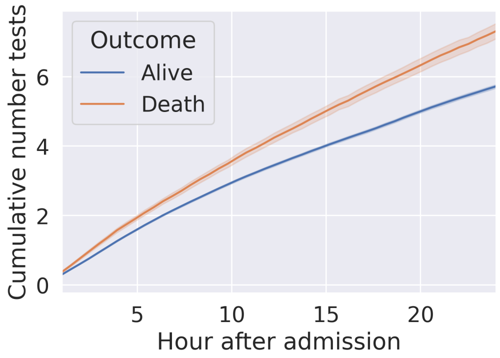

Figure 8: Evolution of the number of tests during the observation period divided by outcome.

Experimental design. For this real-world dataset, patients are split into three groups: 80% for training, 10% for validation and 10% for hyper-parameters selection. The hyper-parameter search consisted of the l2 penalty selection for the logistic regression among $\lambda \in [0.1, 1., 10., 100.]$.

We bootstrapped the test set 100 times and report the mean and 95% confidence bounds.

Tabular results. Table 5 presents the AUC-ROC for each group and imputation strategy. Similarly Table 6 (resp. 7) shows the prioritisation (resp. the false negative rate).

Table 5: AUC-ROC performance divided by group and imputation strategy - Bootstrapped mean (std). Bold indicates the highest AUC-ROC.

	Imputation strategy		
Group	Median	MICE	Group MICE
Black	**0.805** (0.030)	0.781 (0.036)	0.765 (0.037)
Non Black	**0.744** (0.010)	0.740 (0.010)	0.738 (0.010)
Female	**0.735** (0.016)	0.733 (0.018)	0.730 (0.018)
Male	**0.757** (0.016)	0.748 (0.014)	0.744 (0.014)
Public	**0.739** (0.011)	0.726 (0.012)	0.722 (0.013)
Private	0.751 (0.021)	**0.771** (0.018)	0.759 (0.021)
Population	**0.748** (0.011)	0.742 (0.012)	0.738 (0.012)

Table 6: Prioritisation rate divided by group and imputation strategy - Bootstrapped mean (std). Bold indicates the highest prioritisation rate.

	Imputation strategy		
Group	Median	MICE	Group MICE
Black	**0.343** (0.017)	0.323 (0.017)	0.150 (0.013)
Non Black	0.296 (0.004)	0.298 (0.004)	**0.313** (0.005)
Female	**0.328** (0.008)	0.320 (0.007)	0.315 (0.007)
Male	0.280 (0.006)	0.285 (0.006)	**0.289** (0.006)
Public	0.350 (0.006)	0.342 (0.006)	**0.354** (0.007)
Private	0.200 (0.007)	**0.215** (0.007)	0.193 (0.006)
Population	0.299 (0.004)	0.299 (0.004)	0.300 (0.004)

Table 7: False Negative rate divided by group and imputation strategy - Bootstrapped mean (std). Bold indicates the smallest FNR.

	Imputation strategy		
Group	Median	MICE	Group MICE
Black	**0.298** (0.076)	0.314 (0.078)	0.520 (0.081)
Non Black	0.365 (0.016)	0.370 (0.018)	**0.356** (0.019)
Female	0.376 (0.030)	0.378 (0.030)	**0.371** (0.029)
Male	**0.346** (0.027)	0.357 (0.025)	0.367 (0.025)
Public	**0.339** (0.021)	0.351 (0.018)	0.341 (0.020)
Private	0.425 (0.039)	**0.412** (0.039)	0.458 (0.042)
Population	**0.361** (0.019)	0.366 (0.020)	0.367 (0.020)

Table 8: AUC-ROC performance divided by group and imputation strategy - Bootstrapped mean (std). Bold indicates the highest AUC-ROC.

	Imputation strategy with **missing indicators**		
Group	Median	MICE	Group MICE
Black	**0.827** (0.027)	0.817 (0.029)	0.814 (0.029)
Non Black	**0.786** (0.010)	0.785 (0.010)	0.783 (0.010)
Female	0.770 (0.013)	**0.772** (0.014)	0.769 (0.014)
Male	**0.801** (0.013)	0.798 (0.013)	0.796 (0.013)
Public	**0.773** (0.010)	0.770 (0.010)	0.767 (0.011)
Private	0.816 (0.016)	**0.824** (0.016)	0.821 (0.017)
Population	**0.789** (0.008)	0.788 (0.009)	0.786 (0.009)

Table 9: Prioritisation rate divided by group and imputation strategy - Bootstrapped mean (std). Bold indicates the highest prioritisation rate.

	Imputation strategy with **missing indicators**		
Group	Median	MICE	Group MICE
Black	0.317 (0.016)	**0.318** (0.016)	0.194 (0.014)
Non Black	0.298 (0.005)	0.298 (0.005)	**0.309** (0.005)
Female	**0.320** (0.008)	0.314 (0.007)	0.315 (0.007)
Male	0.285 (0.006)	**0.290** (0.006)	0.289 (0.006)
Public	**0.341** (0.005)	0.338 (0.006)	0.339 (0.006)
Private	0.220 (0.006)	**0.225** (0.005)	0.222 (0.005)
Population	0.300 (0.005)	0.300 (0.004)	0.300 (0.004)

Table 10: False Negative rate divided by group and imputation strategy - Bootstrapped mean (std). Bold indicates the smallest FNR.

	Imputation strategy with **missing indicators**		
Group	Median	MICE	Group MICE
Black	**0.224** (0.063)	0.251 (0.065)	0.419 (0.071)
Non Black	0.300 (0.018)	0.287 (0.018)	**0.285** (0.018)
Female	0.298 (0.023)	**0.294** (0.026)	0.311 (0.027)
Male	0.296 (0.025)	**0.272** (0.025)	0.277 (0.027)
Public	0.292 (0.020)	**0.279** (0.018)	0.288 (0.019)
Private	0.302 (0.038)	**0.301** (0.039)	0.321 (0.040)
Population	0.294 (0.017)	**0.283** (0.018)	0.293 (0.018)

Missing Indicators. Similarly, Table 8 presents the AUC-ROC for each group and imputation strategy when missing indicators are added to the regression model. Table 9 (resp. 10) shows the prioritisation (resp. the false negative rate) for the same pipelines. This analysis shows that the missingness patterns are informative of the outcome of interest as adding the missing indicators as regressors improves performance. Note, however, that MICE Group presents lower AUC performance than MICE in Table 8. This observation echos the criticism of adding covariate for a more plausible MAR assumption. Finally, these tables underline how no method consistently outperforms the others across groups and metrics.

Threshold sensitivity. In Section 5.2, we present results for a policy of 30% additional care. As we arbitrarily chose this threshold, we propose to measure how the results vary under two different thresholds: 5% and 50%. Figures 9, 10 and 11 present the results at 5%, 30% and 50% thresholds. First, note that the magnitude of the Δ in prioritisation increases with larger thresholds, but similar trends are observed. This indicates that members of the same group have similar risk scores. Increasing the threshold, therefore, further penalises this whole group. Second, the Δ in false positive rates demonstrates how the choice of imputation is sensitive to the target task. In addition to validating the insights from Section 5.2, this set of experiments demonstrates that the target task may also affect whether an imputation methodology favours or penalises a given group.

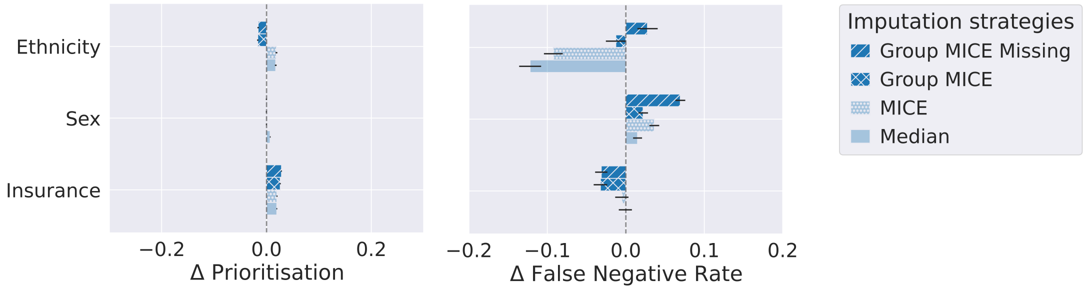

Figure 9: Prioritisation performance gaps Δ across marginalised groups in MIMIC III experiment for 5% additional care. If $\Delta > 0$, the marginalised group has a larger value of the given metric than the rest of the population.

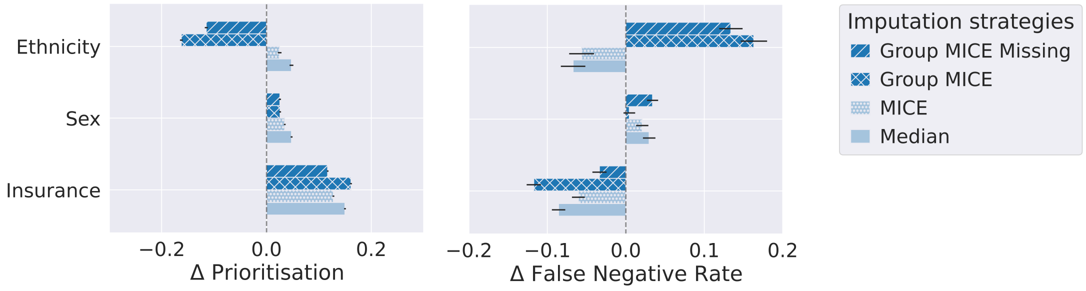

Figure 10: Prioritisation performance gaps Δ across marginalised groups in MIMIC III experiment for 30% additional care. If $\Delta > 0$, the marginalised group has a larger value of the given metric than the rest of the population.

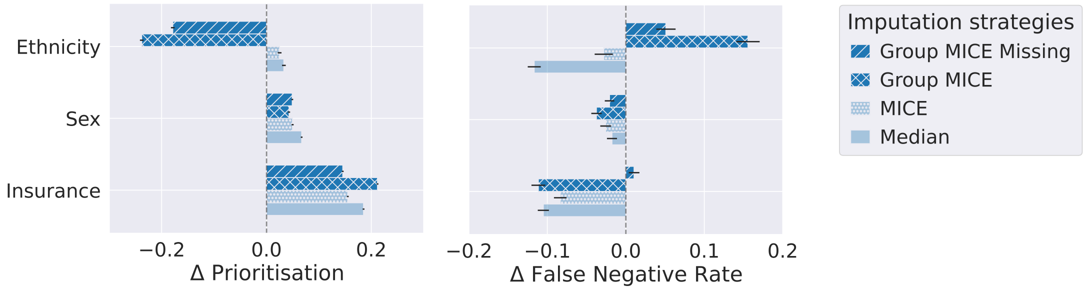

Figure 11: Prioritisation performance gaps Δ across marginalised groups in MIMIC III experiment for 50% additional care. If $\Delta > 0$, the marginalised group has a larger value of the given metric than the rest of the population.
